# Matrix Decomposition of Carbon-Fiber-Reinforced Plastics via the Activation of Semiconductors

**DOI:** 10.3390/ma13153267

**Published:** 2020-07-23

**Authors:** Philippa Ruth Christine Böhnke, Iris Kruppke, David Hoffmann, Mirko Richter, Eric Häntzsche, Thomas Gereke, Benjamin Kruppke, Chokri Cherif

**Affiliations:** 1 Institute of Textile Machinery and High Performance Materials Technology, TU Dresden, Hohe Straße 6, 01069 Dresden, Germany; philippa.boehnke@tu-dresden.de (P.R.C.B.); david.hoffmann@tu-dresden.de (D.H.); mirko.richter@tu-dresden.de (M.R.); eric.haentzsche@tu-dresden.de (E.H.); thomas.gereke@tu-dresden.de (T.G.); chokri.cherif@tu-dresden.de (C.C.); 2 Institute of Materials Science, TU Dresden, Budapester Straße 27, 01069 Dresden, Germany; benjamin.kruppke@tu-dresden.de

**Keywords:** atomic force microscopy, CF, CFRP, depletion, recycling, repair, single filament tensiometry, UV radiation

## Abstract

The present study proposed a novel process for the matrix decomposition of carbon-fiber-reinforced plastics (CFRPs). For this purpose, the influence of ultraviolet (UV) radiation paired with semiconductors on CFRP was analyzed. Then, suitable process parameters for superficial and in-depth matrix decomposition in CFRP were evaluated. The epoxy resin was decomposed most effectively without damaging the embedded carbon fiber by using a UV light-emitting diode (LED) spotlight (395 nm, Semray 4103 by Heraeus Noblelight) at a power level of 66% compared to the maximum power of the spotlight. Using a distance of 10 mm and a treatment duration of only 35–40 s achieved a depth of two layers with an area of 750 mm^2^, which is suitable for technological CFRP repair procedures. In addition to the characterization of the process, the treated CFRP samples were analyzed based on several analytical methods, namely, light microscopy (LM), scanning electron microscopy (SEM), and atomic force microscopy (AFM). Subsequently, the prepared carbon fibers (CFs) were tested using filament tensiometry, single filament tensile tests, and thermogravimetric measurements. All analyses showed the power level of 66% to be superior to the use of 96% power. The gentle (“fiber friendly”) matrix destruction reduced the damage to the surface of the fibers and maintained their properties, such as maximum elongation and maximum tensile strength, at the level of the reference materials.

## 1. Introduction

Lightweight structures offer enormous potential owing to their combination of reduced weight and high stiffness. Furthermore, the corresponding application range is increased, e.g., in the automotive, aerospace, and construction industries, as well as wind energy, sports, and leisure [1,2,3,4,5]. Nevertheless, material prices are high and carbon-fiber-reinforced plastics (CFRPs) leave room for improvement in terms of recyclability and reparability [1,2,6,7,8,9,10]. Currently, there are only very few established mechanical repair methods, and as such, it is common practice to completely replace a damaged component [11,12,13]. Existing mechanical repair processes include the doubler method and the scarf method [14,15,16]. The first method is also called bolted repair and focusses on the removal of the defect via milling. Afterward, the detached part of the composite is repaired with a doubler, which is an additional material that is glued or riveted onto the component. This doubler can be made of various materials, for example, aluminum, titanium, or CFRP. In most cases, riveting is used to attach the doubler to the component. Hence, this method results in a structurally damaged component with an interrupted fiber course, a locally decreased rigidity, and an increased weight. Additionally, for this treatment, a minimum thickness of the component is required. The surface quality and aerodynamic characteristics are also negatively affected. This method is often used for aerospace engineering repairs [12,17]. The scarf method procedure is based on the shaft ratio, whereby the dimension of the milled area around the damage depends on the depth of the defect. Owing to this defined shaft ratio, partially unnecessary damage is done to the component. Moreover, this type of repairing method using glue to refill the milled defect is only warranted up until a defect depth of 1.6 mm. The method also involves high manufacturing costs and is very time-consuming compared to bolted repair [11,12,18]. Both introduced repair methods reduce the final composite strength of the component, thus being unable to restore the original surface qualities.

Another well-known method employs a laser to cut the damaged area out of the component layer by layer. However, this is still a version of the scarf method with a different material removal strategy. The use of the laser allows for more precise cutting but does not present a holistic repair method [19,20,21,22,23,24,25].

A local matrix decomposition of CFRP that allows for locally depleting the matrix of CFRP components without causing negative effects to the reinforcing fibers was investigated earlier [26,27]. However, this treatment is only suitable for specially developed resin systems based on cyanate.

Another approach involves the biochemical depletion of polyester and epoxy resins and was introduced by the Hohenstein Institute (Bönnigheim, Germany). This research aimed at the complete local decomposition of the matrix by microorganisms. The main disadvantage of this approach is the long duration of the process [28].

There is also the option to use a photocatalyst for the degradation process, resulting in a photocatalytic matrix decomposition. Owing to the ability of photocatalysts to absorb light, electron–hole pairs can be generated, whereby a chemical change of the reactants takes place. The chemical composition of the photocatalysts is regenerated after each reaction. Some metal oxides, such as the oxides of vanadium, chromium, titanium, zinc, tin, and cerium, display photocatalytic characteristics. Within a photocatalytic process, the metal oxides are activated by wavelengths in the spectrum between 250 nm and 450 nm but also in the area of infrared (IR) light. Afterward, electrons are elevated from the valence band to the conduction band due to the energy absorbed from the radiation. Within this step, an electron–hole pair is produced. This pair can oxidize the material it is integrated within or lying upon [29,30].

Polymers have the peculiarity of being radically degraded by the destruction of chemical bonds through ultraviolet (UV) light, which results in depolymerization. The energy that is required to break the bonds of most polymers lies between the wavelengths of 290 and 400 nm. Photodegradation arises through the activation of polymer macromolecules via the absorption of photons. These photons break the molecules into smaller parts, thus initiating the decomposition of the polymer. Usually, photodegradation also includes oxidation. The process can be divided into three phases: initiation, propagation, and termination. Within the initiation, photochromic groups in the polymer absorb photons and radicals are formed. In the propagation phase, the radical chain reaction continues and polymer chains are decomposed. The last phase terminates the chain reaction since there are no radical products left [31]. Through photodegradation, the molecular weight changes as a result of the breakup of bonds. This affects the mechanical characteristics and increases the chemical reactivity [32,33].

**Figure 1:**
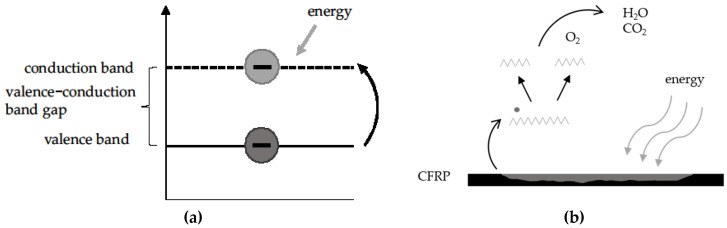
(**a**) Electron jump from the valence band to the conduction band via the addition of energy and (**b**) depletion of the matrix by energy, resulting in the production of water and carbon dioxide. CFRP: Carbon-fiber-reinforced plastic.

In this section, the principle behind radical degradation is explained, which is one possible explanation for the later shown matrix decomposition. As shown in Figure 1a, a catalyst (semiconductor) is activated through energy input, which initiates the radical process.

In this case, matrix decomposition occurs, whereby the fiber remains intact in the composite. This is a theoretically emission-free process with carbon dioxide and water arising as process products (cf. Figure 1b). Additionally, volatile substances are a result of the process. The stimulation of semiconductors is achieved via UV radiation. During the “generation” step an electron from the semiconductor is raised from the valence band into the conduction band. Owing to this electron deficit, radicals are formed. This allows polymer chains to break (i.e., depolymerization) in close vicinity to irradiated semiconductors. This may cause a chain reaction, as long as the energy input is constant and the electron does not jump back into the valence band (recombination) [29,30]. 

According to the literature, this process has not yet been used to recycle fiber-reinforced plastics [34]. Several research groups have analyzed the process and characterized the catalysts used [35,36,37]. In the corresponding papers, TiO_2_, Ni-Cr, ZnO, and Cr_2_O_3+x_ were listed as appropriate semiconductors. They were thermally activated with IR radiation [34,35,36,37]. Previous investigations from our group have also focused on the matrix decomposition of CFRP via the use of IR radiation and semiconductors [38]. 

All conventional repair methods that are presently available have considerable deficits. Although the original damage is repaired, the mechanical processes involved cause great damage to previously intact areas. Additionally, the original composite strength and surface quality cannot be restored after the process. The method involving the photodegradation of polymers for CFRP recycling through activation by IR radiation is also disadvantageous since the required wavelengths lead to temperatures of about 400 °C. This heat negatively affects the whole composite structure.

Within the research presented in this paper, the process of matrix decomposition of CFRP through the stimulation of semiconductors by UV radiation was investigated. Thus, analytical methods and technical textile testing were employed to prove the effectiveness of the process. The study focussed on the effect of the process on the filaments in the CFRP. This effect was determined using analytical methods and single filament tests. The morphology was characterized using light microscopy (LM), scanning electron microscopy (SEM), and atomic force microscopy (AFM), in addition to polished micrograph sections. Using these methods, the structure of treated CFRP samples was analyzed and compared with an intact reference. Moreover, the effects of the process in terms of the marginal areas and filaments embedded in the matrix were investigated. Furthermore, the mechanical properties were evaluated by using a single filament tensile test to characterize the filaments’ maximum elongation and force in comparison to an unprocessed conventional carbon filament. Finally, thermogravimetric analyses (TGAs) were performed on the filaments, pure epoxy resin, and resin combined with the semiconductor. Based on these analyses, it could be established whether the process affected the molecular chains of the filaments. The TGA on the resin revealed how the semiconductor influenced the thermal degradation behavior of the resin.

## 2. Materials and Methods

### 2.1. Materials

Within the project, cerium (IV) oxide (CeO_2_) from Alfa Aesar (Kandel, Germany) was used as the semiconductor. Previously, trials have been made with a range of semiconductors (e.g., titanium dioxide (TiO_2_) from Merck KGaA, Darmstadt, Germany). Within these experiments, CeO_2_ showed the best results in terms of the depleted quantities of the matrix and process parameters. The samples that were used to develop this process were pure epoxy resin samples and CFRP samples (two-layered and four-layered non-crimp fabric (NCF) with symmetric layering (one layer: 0°/90°) and a fiber volume content of about 46–50%). The carbon fiber (CF) used for the NCF was Toray T700SC 50C 12K with a diameter of about 7 µm. The NCF was from Saertex GmbH & Co. KG (Saerbeck, Germany). The used epoxy resin was made with a ratio of 30:100 of RIMH137:RIMR135 from Hexion GmbH, Duisburg, Germany. After the infiltration with a resin transfer molding (RTM) process (cavity height 1.5 mm, pre-heating at 60 °C, pressure ascending from 1–6 bar), the composite samples were cut into square plates with an edge length of 50 mm. Afterward, the mold was heated in the oven to 60 °C and the samples were annealed for 15 h at a temperature of 80 °C.

Furthermore, filaments (CF) without a sizing agent were used. To prepare these samples, a Soxhlet extraction was done. Ethanol was used as the solvent to free the fibers from the sizing agent.

### 2.2. Activation of Semiconductors Using UV Radiation

For the UV spotlight, the Semray 4103 manufactured by Heraeus Noblelight GmbH (Hanau, Germany) was used. The emitting area was constructed out of many light-emitting diodes (LEDs) on a spot area size of 3465 mm^2^, as reported in the datasheet. It had a specific wavelength maximum of 395 nm. The maximum irradiance was 18 W·cm^−2^. While a laser emits electromagnetic waves with high intensity, a sharp wavelength range, and a highly concentrated ray, the UV spotlight Semray 4103 emitted light that was not focused but rather was distributed on the stated area.

The depletion process was conducted in a fume cupboard since the process gases were produced and occupational safety had to be considered.

At first, the samples were prepared by washing them with gentle solvents, and afterward, with deionized water. The dry semiconductor was spread using a strainer and a pattern to generate a precise application. The amount of semiconductor applied corresponded to 1 wt% of the CFRP sample. The sample was placed underneath the UV spotlight with a distance of 10 mm. Then, the radiation source’s power level was set to 66% (equivalent to 11.9 W·cm^−2^) or 96% (equivalent to 17.3 W·cm^−2^) to compare the results of the different performances. The power levels were chosen based on parameter diagrams of the spotlight. Here, the output was calculated based on the distance to the sample and the set output of the spotlight. The treatment lasted about 35–40 s. This duration achieved a depth of two layers with an area of 750 mm^2^. The depth was reached due to the chain reaction of the radicals that spread from their start position. Afterward, the sample had to be mechanically cleaned with a soft brush to remove the semiconductor from the sample.

### 2.3. Methods

#### 2.3.1. Light Microscope

The treated samples were cut into smaller sections, and afterward, embedded into epoxy resin. After this, the sample was tempered and polished. Additionally, images from the surface of the samples were taken using the Scope A.1 Axio from Zeiss (Oberkochen, Germany). The microscope was also used to measure the boundary areas of the treated samples to evaluate the effect of the process around the spotted area.

#### 2.3.2. Atomic Force Microscope

An AFM Tosca 400 by Anton Paar GmbH (Graz, Austria) was used to determine the morphological changes of the samples’ surfaces. For the measurements, the contact resonance amplitude imaging (CR AI) mode was used, which was chosen for the filaments because through this procedure, a precise illustration of the surface topography could be made.

For the AFM measurements, filaments were gently separated from the exposed area. After fixing the fibers on a sample plate, the cantilever was focused on the filament surface in a first step. Then, the tip, which was attached to an actuator unit, was brought close to the sample until contact was made. Subsequently, the tip was excited for the measurement with a frequency of approximately 320 kHz, where the appropriate excitation frequency was determined using excitation frequency sweeps. The software Tosca Analysis 7.4 was used to evaluate the AFM measurements. Small tilt angles were smoothed to be able to display the process-related defects better in the plane image. Two measurements were made for each sample. One as an overview with the dimensions of 5 µm × 10 µm and the second one as a detailed image with dimensions of 2 µm × 2 µm.

#### 2.3.3. Scanning Electron Microscope

The surface morphology of the CFs was characterized using SEM. To this end, manually cut fiber segments were glued on a sample holder with a carbon table. Secondary electron images were taken using a Philips ESEM XL 30 SEM (Amsterdam, Netherlands) working in high vacuum mode with a 3 kV acceleration voltage and a 6.5 mm working distance. Representative images at magnifications of 500× and 10,000× were selected from the image series with magnifications between 125× and 40,000×.

#### 2.3.4. Tensiometry

Through the use of standard DIN 55660-2, the surface energy of the detached CFs was measured. At first, the filaments had to be prepared. This occurred by detaching them from the samples with tweezers, and afterward, gluing them onto special sample holders. Five filaments were fixed on a microscope slide.

The tensiometry itself was accomplished using a Krüss Force Tensiometer K100 (Hamburg, Germany). This device used the comb method. The measurement was performed with deionized water (surface tension at 23 °C, γ_lv_ = 72.8 mN·m^−1^) and diiodmethane (>99%, Sigma-Aldrich Chemie Gmbh (Darmstadt, Germany), surface tension at 23 °C, γ_lv_ = 50.8 mN·m^−1^) separately. Both fluids were necessary to specify the polar and disperse parts of the surface energy. Before the measurement could be started, the diameter, which was previously determined using the light microscope Scope A.1 Axio manufactured by Zeiss (Oberkochen, Germany), was registered in the software Advance that was used by the device to record the test. During the measurement, the filaments were dipped into the liquid at a specific speed. The detached fibers of the treated samples were measured in comparison to the reference fiber that was not embedded and a reference fiber freed from the sizing agent. The measurement was performed three times for each liquid.

The evaluation was made based on the surface free energy (SFE) method. The determination was carried out according to Owens, Wendt, Rabel, and Kaelble [39,40,41] (Equation (1)):

(1) $$\frac{\left(1+\cos\theta \right)\cdot \sigma_{l}}{2\cdot \sqrt{\sigma_{l}^{D}}}=\sqrt{\sigma_{s}^{P}}\cdot \sqrt{\frac{\sigma_{l}^{P}}{\sigma_{l}^{D}}}.$$

The total surface free energy was calculated using the contact angles (*θ*). Furthermore, the polar (*σ^P^*) and disperse (*σ^D^*) terms of the surface free energy were determined. Here, the indices *l* and *s* are important because they describe the solid and liquid states, respectively, i.e., the test liquid and the filament surface.

The average diameters of the reference and the detached filaments (66% power) was 7.0 µm and 7.35 µm, respectively. The filaments detached from the sample treated with 96% power and a cleaning process had an average diameter of 7.67 µm.

#### 2.3.5. Single Filament Tensile Test

The test was done using standard ISO 11566 for CFs, where the device used for the measurement was the Favimat manufactured by Textechno Herbert Stein GmbH & Co. KG (Mönchengladbach, Germany). During this test, single filaments were detached from the samples. For each filament type, at least 50 measurements were made. Some detached fibers of the treated samples were measured in comparison to a reference fiber that had not been embedded and a reference fiber that was freed from the sizing agent. For the clamping, the combination of Vulkollan and hard rubber was used with a clamping force of 5 bar. Furthermore, the parameters were a preload of 0.5 cN·tex^−1^, the clamping length was 20 mm, and the test was carried out with a speed of 10 mm·min^−1^.

#### 2.3.6. Thermogravimetric Analysis (TGA)

TGA is an analytical method in which the change of bulk of a sample is measured as a function of temperature and time. For this paper, the TGA Q500 from TA Instruments (New Castle, DE, USA) was used. Within this test, there was a temperature field of 30 °C to 800 °C under atmospheric conditions. Some detached fibers of the samples treated with power outputs of 66% and 96% were measured in comparison to a reference fiber that had not been embedded and filaments that were freed from the sizing agent.

Additionally, temperature measurements were made. The first recorded data about the temperature development was made without any sample underneath the UV radiator. The second measurement recorded the temperature profile of a process implemented with powers of 96% and 66%.

## 3. Results

### 3.1. Morphological and Surface Characterization

#### 3.1.1. Atomic Force Microscopy

**Figure 2:**
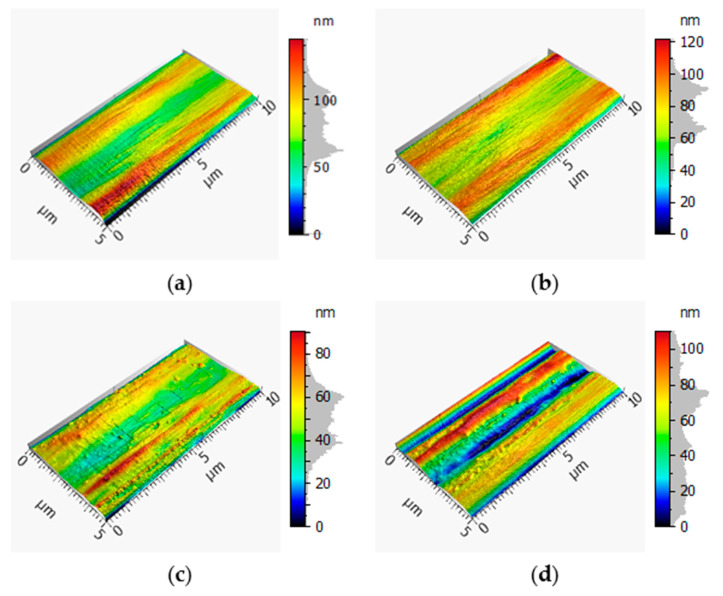
3D, 5 µm × 10 µm surface profile of (**a**) the reference fiber, (**b**) the reference without the sizing agent, (**c**) a fiber detached with a power output of 66% and (**d**) a fiber detached from the treated sample with a power output of 96%.

The following AFM images show the surface of the reference fiber and the separated fiber treated with a UV spotlight at 66% and 96% power. Within the records, two elevations with a gap in between can be observed. The width of the gap between these elevations was about 2.5 µm. The elevations were measured using the AFM. The fiber had a round surface that was depicted as a planar surface in the AFM images. A smoothing algorithm was used to create these records. Nevertheless, a change of the surface was shown after the treatment, especially after the use of 96% power compared to the more gentle conditions at 66% power (Figure 2c,d).

In Figure 2a, impurities cannot be seen on the surface; however, as is typical for CF, striation structures occurred on the filament’s surface [42]. Figure 2b shows the record of a reference filament without a sizing agent. Here the surface was less smooth than the reference surface. Additionally, there were no special morphologies. Striations were present on the surface of the filament treated with an output of 66%; this can be observed in Figure 2c. In comparison to the reference pictured in Figure 2a, the striations from the detached filament were narrower. Furthermore, impurities can be seen on the filament’s surface. Figure 2d exhibits an AFM image of separated filament samples treated with a power output of 96%. The record in Figure 2d shows two deep recesses, and subsequently, two elevations. Furthermore, there were impurities on the filament’s surface. 

**Figure 3:**
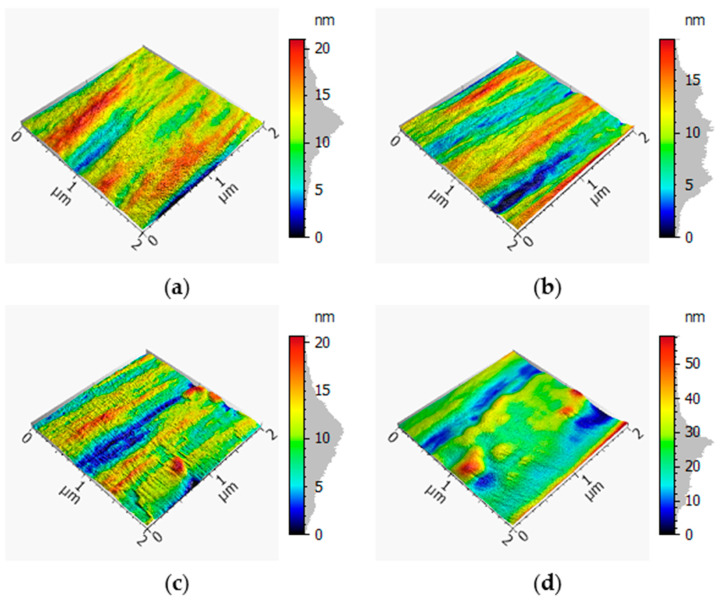
3D, 2 µm × 2 µm surface profile of (**a**) the reference fiber, (**b**) the reference without the sizing agent, (**c**) a fiber detached with a power output of 66% and (**d**) a fiber detached from the treated sample with a power output of 96%.

Figure 3 provides more detailed records of the filaments, where Figure 3a,b depict both references and show similar surfaces. The filament detached from a sample treated with a power output of 66% (Figure 3c) displayed a rougher surface with more elevations and recesses, but was quite comparable to the reference fiber without the sizing agent, as seen in the arithmetic heights of 10.80 ± 0.28 nm and 10.84 ± 3.76 nm, respectively (Table 1). Figure 3d shows a filament detached from a sample treated with a power output of 96% and presented the smoothest surface of all four records. The elevations and recesses were detected but the transition was smooth.

Within Table 1, the average arithmetic height deviation and the maximum height of the samples are shown. The filament detached from the sample treated with 96% power showed the highest values, followed by the reference. The sample treated with a power output of 66% had the lowest value for the maximum height, together with the reference without the sizing agent. The average deviation was smaller for the reference without the sizing agent compared to the reference. The lowest average deviation occurred with the filament detached from a sample treated with a power output of 66%. The sample treated using 96% power showed the highest average deviation.

#### 3.1.2. Scanning Electron Microscopy

**Figure 4:**
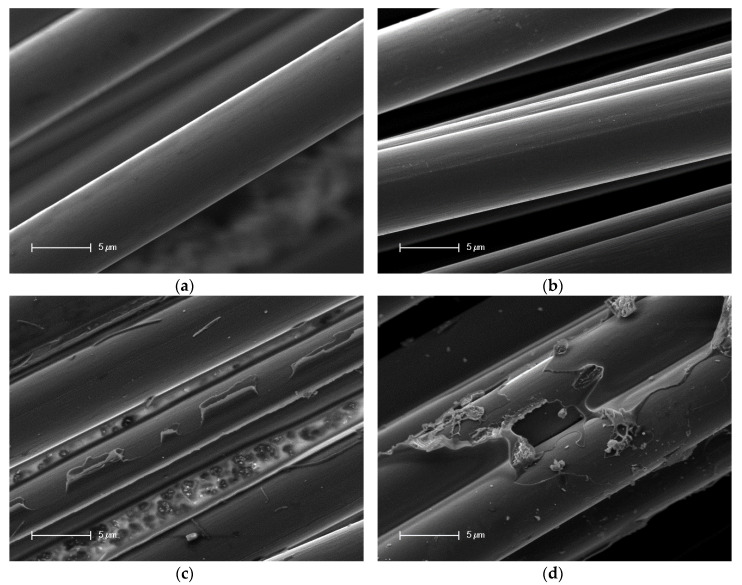
Scanning electron microscopy (SEM) images of the filaments: (**a**) reference, (**b**) reference without the sizing agent, (**c**) detached and treated with 66% power, and (**d**) detached and treated with 96% power.

**Figure 5:**
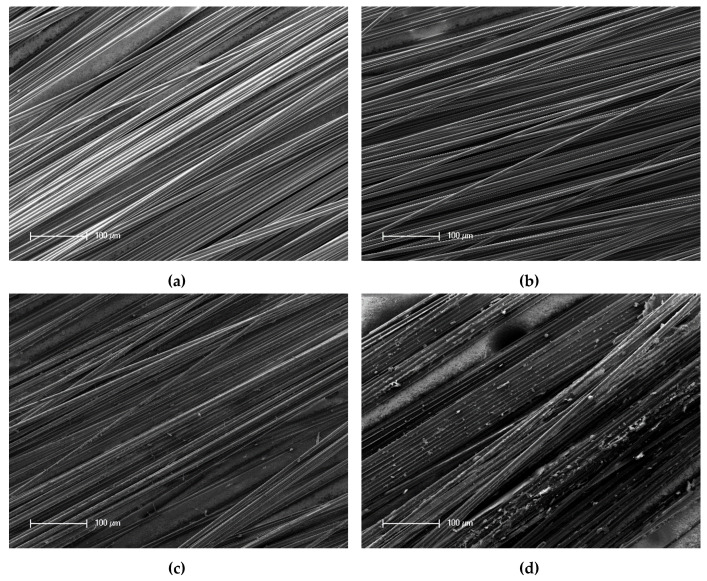
Scanning electron microscopy (SEM) images of the filaments: (**a**) reference, (**b**) reference without the sizing agent, (**c**) detached and treated with 66% power, and (**d**) detached and treated with 96% power.

The following pictures in Figure 4 and Figure 5 show the SEM records of the filaments. The reference is depicted with and without the sizing agent, as well as the detached filaments treated with output powers of 66% and 96%.

In Figure 4a, the reference filament is depicted. The surface of the fiber was very smooth and showed no impurities or damage. Figure 4b shows the reference without the sizing agent. Small impurities were visible on the surface. Nevertheless, the surface showed no damage. Figure 4c shows the filaments detached from a sample treated with a power output of 66%. Impurities and leftover matrix were present on the surface. Additionally, between the filaments, the holes generated by the process were visible. The last picture shows the filaments treated beforehand with a power output of 96%. The surface was almost completely covered by leftover matrix. Furthermore, it had impurities.

Figure 5 shows a general overview of the filaments. In comparison with Figure 5a,b, the impurities and leftover matrix are shown in Figure 5c,d, in which Figure 5d showed much more of these.

#### 3.1.3. Light Microscopy

The boundary areas of the samples treated with power outputs of 66% and 96% were found using LM. The sample treated with the higher output showed a larger boundary area, with many intermediate process products surrounding the treated area. The sample treated with a power output of 96% had a boundary area width of 1328 ± 37 µm. The sample treated with 66% power had a boundary area of 568 ± 34 µm. This resulted in a reduction of 57% of the boundary area by using 30% less power for the process.

**Figure 6:**
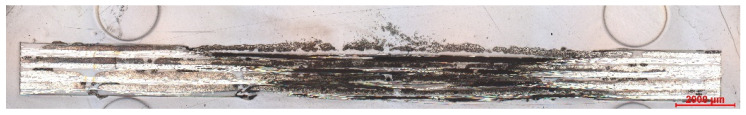
Polished micrograph section of a four-layered carbon-fiber-reinforced plastic (CFRP) sample treated with 66% power.

Figure 6 shows a polished micrograph section of a four-layered and treated sample of CFRP.

In Figure 6, it can be observed that the process reached all layers of the sample. It was treated on both sides using the process described in Section 2.2. The treated area is the region in black. In the middle of the sample, the treated width got smaller. The chain reaction of the depletion of the matrix on the surface continued inside the sample. However, the treated area got smaller the deeper it penetrated. Furthermore, some of the rovings unbound from the composite were visible on top of the sample. With the set process parameters, the four-layered CFRP sample could be completely penetrated and subsequently freed from the matrix.

### 3.2. Mechanical Characterization

#### Single-Fiber Tensile Test

**Figure 7:**
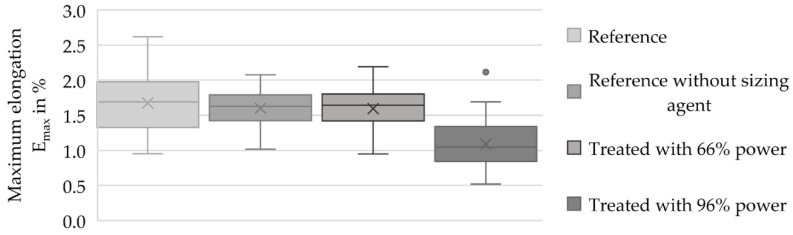
Boxplot diagram with the maximum elongation results (E_max_ in %) of the filament references and the treated and detached filaments.

**Figure 8:**
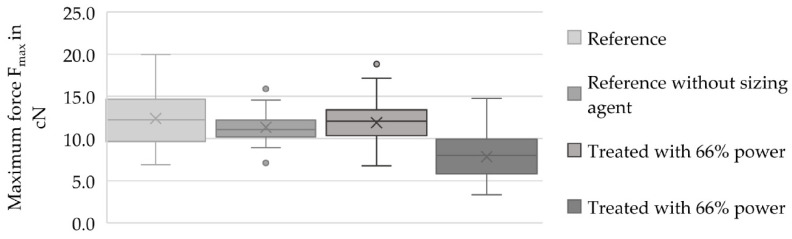
Boxplot diagram with maximum force results (F_max_ in cN) of the filament references and the treated and detached filaments.

Within this test, the maximum force and maximum elongation of the filaments were compared. Figure 7 shows the maximum elongation of the filaments detached from the samples and Figure 8 shows the maximum force used to break the filaments detached from the samples.

Figure 7 exhibits the results of the tensile test regarding the maximum elongation. The reference had an average maximum elongation of 1.68 ± 0.41%. The detached fiber (treated with 66% power) exhibited an average maximum elongation of 1.59 ± 0.29%, which was 94.64% of the reference’s maximum elongation. The filaments treated with 96% power had a value of 1.09 ± 0.34%, which was 64.88% of the reference’s maximum elongation. The reference freed from the sizing agent had a maximum elongation of 1.60 ± 0.26%; this value corresponded to 95.24% of the reference’s maximum elongation.

Regarding the maximum forces shown in Figure 8, the average maximum force of the reference was 12.36 ± 3.30 cN. The detached fiber (treated with 66% power) had an average maximum force of 11.87 ± 2.74 cN, which was 96.04% of the reference’s average maximum force. The sample that was treated with a power output of 96% had an average maximum force of 7.85 ± 2.52 cN, which was 36.49% less than the reference. Additionally, it was 33.87% less than the sample treated with a power output of 66%. The reference that was freed from the sizing agent showed a value of 11.32 ± 1.90 cN.

### 3.3. Others

#### 3.3.1. Thermogravimetric Analysis

**Figure 9:**
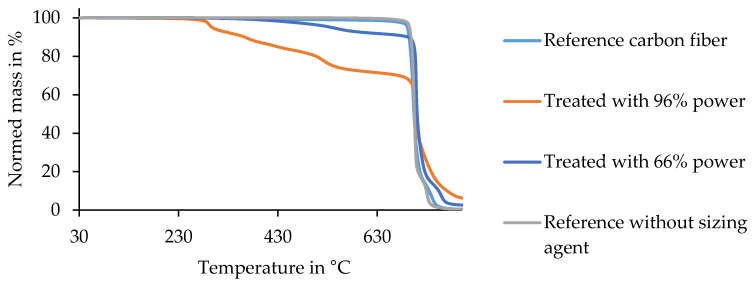
Thermogravimetric analysis of the detached fibers from treated samples in contrast to the fiber reference.

**Figure 10:**
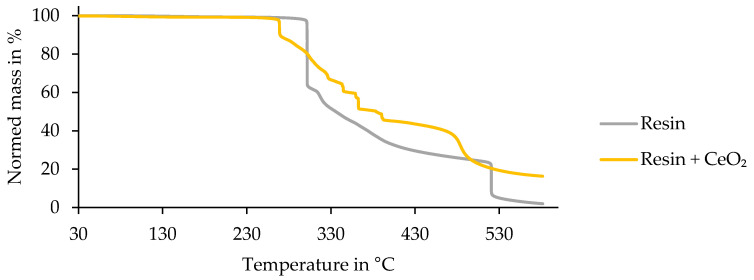
Thermogravimetric analysis of pure epoxy resin and pure epoxy resin and epoxy resin combined with the semiconductor CeO_2_.

Figure 9 depicts the TGA results of the filaments. Here, a thermally induced depletion of the fibers and matrix occurred. This analytical measurement method was used to depict how the depletion process affected the fibers and how the thermally induced photocatalytic process appeared. Within Figure 10, the curves of the TGA of pure resin and resin combined with CeO_2_ are shown.

Within Figure 9, one curve shows the mass loss from the filament reference under the influence of temperature and time. The graph shows the highest loss of mass at a temperature of 702 °C, which can also be found in the literature as the degradation temperature [43]. Here, this involved a reduction of mass of around 85%. The reference was completely decomposed at a temperature of 783 °C. The reference freed from the sizing agent is also depicted in this graph. It showed nearly identical progress to the reference. The next curve shows the results of the filaments treated with a power output of 66%. Here, the first substantial mass loss of 7.7% showed up at a temperature of 620 °C. The most extensive mass loss of around 79% occurred at 709 °C. At 884 °C, there was still some mass of the detached filaments left. The results of the filaments treated with a power output of 96% are shown in the last curve. The mass loss started at 301 °C and finished with some leftover mass at 582 °C. Finally, for this sample, the mass leftover was 1.95% compared to the initial mass.

The sample of pure epoxy resin combined with the semiconductor CeO_2_ showed decomposition of the resin (matrix) that began at a lower temperature of 268 °C compared with pure epoxy resin. There was a recorded mass loss of 11.25%. Afterward, the mass loss continued in smaller steps of about 6–10%. At a temperature of 581 °C, the measurement ended with a leftover mass of 16.31%. Usually, the degradation temperature of resin lies at a temperature of about 300 °C [44].

**Figure 11:**
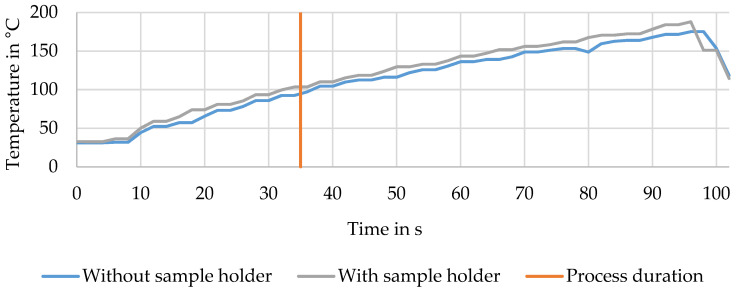
Temperature development underneath a UV radiator with and without the sample holder.

**Figure 12:**
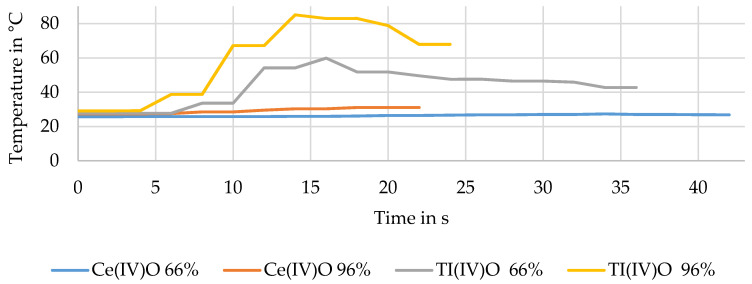
Temperature development underneath a UV radiator during the process.

Figure 11 shows the temperature evolution underneath the UV radiator without and with a sample holder in the test set up. Figure 12 exhibits the temperature evaluation during a process implemented with powers of 96% and 66% and two different semiconductors onto the CFRP.

Between the two curves is a small temperature difference, where the sample was 9.39 K warmer on average with the sample holder underneath. Furthermore, it was shown that over 100 s, a maximum temperature of about 180 °C was reached. The orange vertical line marks the average process duration; the temperature reached lay at about 100 °C.

Figure 12 shows the temperature development during the UV heating process. The dashed line depicts the course of the process implemented with a power of 66% and the black line shows the process with 96% power. Both temperature graphs lay underneath a temperature of 40 °C. For comparison, trials with TiO_2_ are depicted as well. The temperature developments for processes implemented with 96% and 66% powers are shown. Both curves lay above the curves for CeO_2_. The maximum temperature reached by TiO_2_ was about 80 °C. However, the difference between both oxides shows the effectiveness of CeO_2_, which led to the final choice of the semiconductor.

#### 3.3.2. Tensiometry

In Table 2, the results from the single-fiber tensiometry are shown. The analysis was done for the filament reference, filament reference with no sizing agent, and the detached filaments.

The total surface free energy of the reference was 55.37 ± 6.54 mN·m^−1^. The polar part was 11.06 ± 3.22 mN·m^−1^ and the disperse part was 44.32 ± 3.32 mN·m^−1^. The filaments freed from the sizing agent had a total SFE of 40.98 ± 4.11 mN·m^−1^; the polar part of the energy claimed 14.38 ± 2.16 mN·m^−1^ of it. This resulted in a disperse part of 26.59 ± 1.95 mN·m^−1^. Consequently, these filaments had about 74.01% of the reference’s total surface free energy. These SFE values for untreated CFs can also be found in the literature [45,46]. The detached filaments treated with a power output of 66% had a value of 54.60 ± 21.89 mN·m^−1^ for the SFE, where the polar part was 13.20 ± 11.33 mN·m^−1^ and the disperse part was 41.39 ± 10.65 mN·m^−1^. The detached filaments had 98.6% of the reference’s total SFE. Compared to the filaments freed from the sizing agent, there was a percentage similarity of 133.24%. The total SFE of the detached filament treated with a power of 96% was 30.50 ± 18.72 mN∙m^−1^ and was divided into a polar part of 5.97 ± 5.92 mN∙m^−1^ and a disperse part of 24.53 ± 12.80 mN∙m^−1^. These values corresponded to 55.08% and 91.97% of the total SFE of the reference and the reference freed from the sizing agent. Furthermore, the contact angles can be seen in Table 2. The values of the reference freed from the matrix corresponded well to the literature values [47].

## 4. Discussion

### 4.1. Morphological Characterization

It was shown in Figure 6, that the matrix was decomposed by the process of stimulated semiconductors through a UV spotlight. Furthermore, all layers of the sample were depleted of the matrix. Compared to the description of the records from the LM and the measurements, there was still a boundary area where the matrix was not removed completely. In contrast to the sample that was treated with a power output of 96%, the sample treated with 66% power had a much narrower border area, where an improvement of 57% was reached. Additionally, the polished micrograph section showed black areas around the sample. This was a result of air inclusions within the embedded sample.

Through the AFM records exhibited in Figure 2 and Figure 3, it was shown that striations were present, even after treatment with a power output of 66%. The filaments detached from a sample treated with a power output of 96% did not show these special morphologies, as observed in Figure 2d. The treatment caused the fiber surface to be smoothed, as seen in Figure 3d. Additionally, the sample in Figure 2d showed deep recesses on the surface, which indicates that the filament was harmed by the treatment. Furthermore, both filaments that were detached from a treated sample showed elevations, which suggests that there was leftover matrix on the surface. However, the images of the reference and the reference without the sizing agent shown in Figure 3 looked very similar and did not show any impurities or damage. The record of the filament detached from a sample treated with a power output of 66% had a rougher surface, which also indicates that there was leftover matrix on the surface. The same observation could not be made in Figure 3d because at first sight, the surface looked very smooth. However, through the height recordings shown in Table 1, the elevations were much lower for the detached filaments from the sample treated with 66% power, which led to the conclusion that the process cleaned the surface and impurities were eliminated. The filament reference had a higher maximum height, which was a result of the adhesive layer, which accumulated in the striations of the filaments. After the treatment with the semiconductors and the UV radiation, this adhesive layer was removed and a different height profile appeared. Here, the detached filaments treated with a power output of 66% showed a topography with lower maximum heights and lower deviation values compared to the samples treated with a power output of 96%.

Within the SEM pictures, it could be seen that the filaments that were treated with a power output of 66% had a much cleaner surface. Additionally, it had very few matrix residues, especially in contrast to the filaments treated with a power output of 96%, as shown in Figure 5d. This means that through the treatment with a lower output of UV radiation, the filaments were getting cleaner and further processing was made easier.

### 4.2. Mechanical Characterization

The single-fiber tensile tests showed a loss of 3.96% of average maximum force and 5.36% of average maximum elongation relative to the preferred variant with a power output of 66% within the process compared to the reference (cf. Figure 7 and Figure 8). This loss of force could have been caused by the removal procedure of the fiber, as well as the loss of the adhesion layer or sizing agent. The loss of the maximum elongation could have resulted from the process and the cleaning afterward. According to the AFM records in Figure 2, the filaments showed a small amount of damage on the surface, which possibly reduced the mechanical properties. Furthermore, the SEM records in Figure 4c showed leftover matrix between the filaments. This was also a reason for the better elongation value of the filaments treated with a power output of 66%. Consequently, this procedure should be optimized for further investigations because this small amount of damage produces weak spots and premature failure. Additionally, the boxplots in Figure 7 and Figure 8 show that the samples treated with a power of 66% had better mechanical behavior than the ones treated with a power output of 96%. This indicates that the higher power of the UV radiation damaged the filaments. The lower output generated a gentler process for the filaments and hence promising mechanical values.

### 4.3. Other Characterization

The results of the TGA, exhibited in Figure 9 and Figure 10, showed that the process influenced the detached filaments from the treated samples. Nevertheless, the treatment with a UV spotlight power output of 66% caused a mass loss near the reference’s one, which indicated the complete removal of the epoxy resin of the matrix. The TGA of the epoxy resin showed that with the semiconductor, the matrix was decomposed at a lower temperature than without the semiconductor, where the reduction was 30 K. This had a positive effect overall on the process as the temperature was lower and the fiber was influenced less. Furthermore, this result led to the conclusion that the semiconductor was not consumed through the process and worked as a catalyst. In contrast to the pure epoxy resin with a leftover mass of 1.95%, the combination of resin and semiconductor still had a leftover mass of 16.31%. The amount of semiconductor was calculated to be 14 wt%. Compared to the process using IR radiation, which had temperatures of about 350–500 °C, the treatment with the UV spotlight showed a maximum temperature of 180 °C (cf. Figure 11) [38]. Inter alia, this was caused by the short duration of the samples underneath the spotlight and the use of CeO_2_ as the oxide (cf. Figure 12).

The fiber tensiometry (cf. Table 2) showed that the surface free energy of the detached filaments treated with a power output of 66% was nearly the same (98.61%) as that of the reference. This indicates that the treatment with a power output of 66% was gentler to the filaments and the surrounding matrix. In contrast, the filaments treated with 96% power had 91.97% of the surface free energy of the filaments that were freed from the sizing agent. This indicates that the filaments treated with a higher output had less or no sizing agent on their surface. However, this was in contrast with the results from the SEM. There, it was shown that the leftover matrix, and consequently, the sizing agent was still on the surface. This means that the high output of the UV radiation had a negative influence on the fiber’s surface and did not satisfactorily expose the filaments.

## 5. Conclusions

The process developed in the presented study could free two layers of CFRP from a matrix in only one process step. Additionally, the parameters chosen for the process showed mostly auspicious behavior in the case of the filaments embedded in the composite. Morphologically, the detached filaments treated with a power output of 66% had positive results, as seen in the LM, AFM, SEM, and polished micrograph sections. Additionally, the results of the filament tensiometry were very encouraging since the properties of the detached fibers were very similar to those of the reference. In terms of the mechanical characterization, only a negligible difference was detected between the detached fibers of treated with 66% power and the reference.

These results led to the conclusion that the described process provided encouraging results in terms of morphology, tensiometry, and mechanical behavior when using a power output of 66%. Potentially, the process can be adapted to other fiber-reinforced plastics as a repair or recycling method with filaments remaining intact. In addition, this process should be developed further for mobile applications with a smaller spotlight and different deposition methods for the semiconductor. A fast and non-destructive repair method for CFRPs is urgently required. Until now, there have only been a few mechanical repair methods available on the market. Therefore, further studies will involve adjustments of the process parameters regarding the composition and thickness of CFRPs, as well as new application methods for sizing agents for the treated area. This is expected to increase the adhesion between reinforcing fibers in the treated area and the new matrix, yielding a completely novel approach for CFRP repair.

With this new repair concept for CFRP structures, raw material could be saved in the future. Furthermore, small damage and only one-side-accessible damage can be easily repaired such that a replacement of the whole component is unnecessary.

## Figures and Tables

**Table 1:** Arithmetic average height and maximum height of the atomic force microscopy (AFM) samples.

Filament Sample	Arithmetic Average Height Deviation r_a_ in nm	Maximum Height r_z_ in nm
Reference CF	15.75 ± 4.17	116.50 ± 40.31
Reference CF without sizing agent	10.84 ± 3.76	95.00 ± 37.69
Treated with 66% power	10.80 ± 0.28	95.10 ± 6.51
Treated with 96% power	19.40 ± 5.88	123.25 ± 12.69

**Table 2:** Contact angles for the surface free energy (SFE) measurements.

Filament Sample	SFE Total in mN·m^−1^	SFE Disperse in mN·m^−1^	SFE Polar in mN·m^−1^	Contact Angle Water in °	Contact Angle Diiodomethane in °
Reference	55.37 ± 6.54	44.32 ± 3.32	11.06 ± 3.22	59.59 ± 5.95	6.88 ± 13.57
Reference without sizing agent	40.98 ± 4.11	26.59 ± 1.95	14.38 ± 2.16	66.07 ± 3.15	63.45 ± 3.40
Detached + 66% power	54.60 ± 21.98	41.39 ± 10.65	13.20 ± 11.33	57.44 ± 19.50	36.35 ± 22.45
Detached + 96% power	30.50 ± 18.72	24.53 ± 12.80	5.97 ± 5.92	101.36 ± 31.86	49.07 ± 34.99
